# How Can We Prevent Postoperative Kyphosis in Cervical Laminoplasty?

**DOI:** 10.3390/medicina62010058

**Published:** 2025-12-28

**Authors:** Efecan Erisken, Selin Bozdag, Ismail Ertan Sevin, Hasan Kamil Sucu

**Affiliations:** Department of Neurosurgery, Ataturk Training and Research Hospital, Izmir Katip Celebi University, 35620 Izmir, Turkey; efecaneriskenn@gmail.com (E.E.); selin.bzdg@gmail.com (S.B.); seviner67@yahoo.com (I.E.S.)

**Keywords:** cervical, laminoplasty, lordosis, kyphosis, facet disturbance, alignment

## Abstract

*Background and Objectives:* This study aimed to evaluate changes in cervical sagittal alignment after open-door laminoplasty and identify any specific preventable risk factors associated with postoperative kyphotic deformity. *Materials and Methods:* We retrospectively reviewed patients who underwent open-door laminoplasty for degenerative cervical stenosis between 2018 and 2021. Radiological assessment included pre- and postoperative C2–C7 Cobb angles, cervical alignment categories (lordosis, straight, sigmoid, kyphosis), and K-line status. Early postoperative CT scans were analyzed for lamina fractures and facet joint disturbances. Clinical and demographic data, as well as surgical variables such as C3 involvement, were also recorded. *Results:* Among 78 patients with available pre- and postoperative MRI images (mean age 56.5 ± 11.2 years; 42.3% female), the mean cervical lordosis decreased significantly from 8.78 ± 13.75° to 6.49 ± 13.82° (*p* = 0.024). Loss of lordosis was strongly associated with facet disturbance at the cranial-most operated level (*p* = 0.036), inclusion of C3 in laminoplasty (*p* = 0.031), and cranial-most lamina fractures (*p* = 0.004) in univariate analyses. However, in the multivariate logistic regression model, only the uppermost facet disturbance was identified as the independent risk factor for postoperative kyphotic change (OR 4.62, *p* = 0.039). C3 involvement and lamina fracture lost significance after adjustment, likely reflecting collinearity with facet injury at the cranial level. Other demographic or technical variables were not found to be statistically significant predictors. *Conclusions:* Postoperative sagittal alignment after laminoplasty is influenced by surgical complications at the cranial levels. A novel predictor—uppermost facet disturbance—emerged as a significant contributor to loss of lordosis. Preservation of these structures represents a practical strategy to reduce postoperative kyphotic drift. Prospective multicenter validation of the present study’s findings is warranted.

## 1. Introduction

Cervical spondylotic myelopathy (CSM) is the most common cause of spinal cord dysfunction in adults, resulting from progressive degenerative changes that lead to spinal canal narrowing and chronic cord compression. Clinically, CSM manifests with gait disturbance, hand clumsiness, sensory deficits, and upper motor neuron signs. Surgical decompression is the mainstay of treatment for patients with moderate to severe or progressive symptoms. Among posterior decompression techniques, cervical laminoplasty has gained wide acceptance as a motion-preserving procedure that effectively decompresses the spinal cord while avoiding the complications associated with fusion surgery [1,2].

Laminoplasty is a posterior surgical method, which directly decompresses the posterior compressions of the spinal canal while indirectly decompressing the anterior compressions in the lordotic cervical spine via posterior displacement of the spinal cord.

To some extent, laminoplasty inherently damages the posterior musculoligamentous complex and, thus, may cause loss of cervical lordosis over time. It has been reported that approximately 10–15% of patients develop cervical kyphotic deformity after laminoplasty [3]. Once kyphotic deformity develops, the indirect anterior decompression achieved by spinal cord retraction away from the corpus in the early postoperative period can be lost, with the spinal cord again becoming compressed by anterior structures (e.g., posterior surface of the corpus, disk, osteophyte, OPLL).

The preservation of cervical lordosis is considered a prerequisite for positive long-term patient outcomes [4]. Previous studies have reported a relatively poor prognosis for laminoplasty patients who develop kyphosis after surgery [5,6,7]. In addition to preventing re-compression of the spinal cord, maintaining cervical lordosis is crucial for maintaining forward gaze; furthermore, any disruption of lordotic alignment in the cervical spine results in pain and functional loss. Although laminoplasty has been performed for decades as a relatively simple and effective method to treat cervical stenosis, some surgeons prefer anterior or posterior fusion surgery due to the potential risk of postoperative cervical lordosis loss associated with laminoplasty. Although the development of kyphosis after laminoplasty should be prevented whenever possible, the risk factors of kyphosis have not yet been clearly revealed. Although several authors have suggested that parameters such as the inclusion of C3 level in laminoplasty lead to the development of cervical kyphosis, the development of kyphosis appears to be multifactorial [8,9]. The aim of this study is to determine the drivers of changes in cervical lordosis by comparing the pre- and postoperative radiological examinations of patients who underwent cervical laminoplasty to reveal which features are different in patients who developed cervical kyphosis compared with those who did not. Accordingly, modifications in laminoplasty indications and technique can be tailored based on patient-specific characteristics, thereby contributing to better outcomes for patients with cervical stenosis.

## 2. Materials and Methods

This retrospective study was approved by the Ethics Committee of Izmir Katip University (date: 21 April 2022, number: 0191). The clinical archives were retrospectively reviewed for patients who underwent the open-door laminoplasty procedure at the Neurosurgery Clinic of Izmir Ataturk Training and Research Hospital within the period from 1 January 2018, to 31 December 2021.

Inclusion criteria included patients who underwent an open-door laminoplasty procedure for degenerative cervical stenosis.

Exclusion criteria were previous anterior cervical surgery, having a cervical spinal anomaly such as Klippel–Feil Syndrome, laminectomy, and/or posterior instrumentation in addition to laminoplasty. Furthermore, we excluded patients without images in the same modality, taken within one month before surgery and at least one month after surgery, and in which the postoperative change in lordosis angle could be determined.

### 2.1. Surgical Procedure

**Figure 1:**
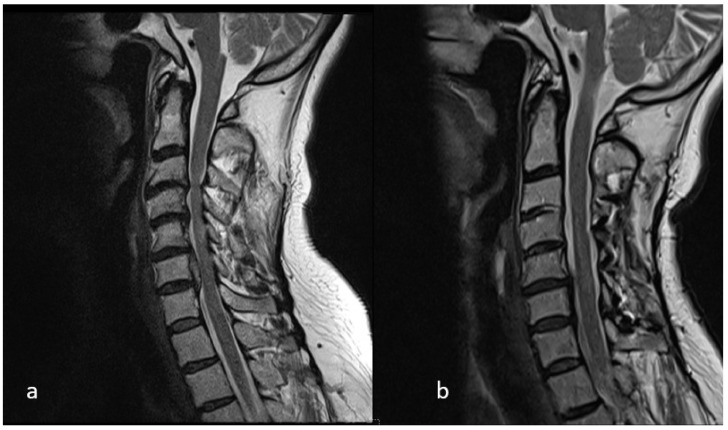
The levels selected for laminoplasty were determined preoperatively based on the extent of spinal cord compression identified on MRI: (**a**) based on the preoperative MRI, it was decided to perform laminoplasty at the C3–C4–C5–C6 levels; (**b**) postoperative MRI showing that the canal had relieved.

**Figure 2:**
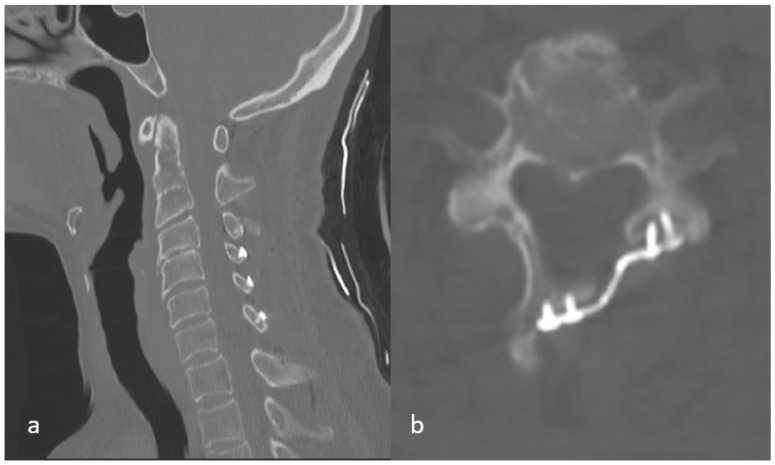
Postoperative CT scan: (**a**) in the sagittal image, the canal has been widened with the help of mini plates; (**b**) in the axial image, the lamina has been lifted with the help of screws fixed to the lateral mass and lamina.

After induction of general anesthesia, the patient was positioned prone on a radiolucent table with the head secured in a Mayfield three-pin head holder. A midline incision was made, and subperiosteal dissection was carried out to expose the laminae, spinous processes, and medial aspects of the facet joints from C2 to C7. The levels selected for laminoplasty were determined preoperatively based on the extent of spinal cord compression identified on MRI (Figure 1). On the open side, a complete trough was created along the junction of the lamina and lateral mass; meanwhile, on the contralateral side, an incomplete trough was fashioned to serve as a hinge. The laminae were then gently opened in an open-door fashion. Mini-plate (Ramed, İzmir, Türkiye) was applied, with the cut edge of the lamina seated into the laminar shelf of the plate and the lateral portion secured onto the lateral mass. Fixation was achieved using two 7 mm screws for the lateral mass and two 5 mm screws for the opened lamina (Figure 2).

### 2.2. Independent Variables

#### 2.2.1. Preoperative Demographic Independent Variables

1. Age
2. Sex

#### 2.2.2. Preoperative Radiological Independent Variables

**Figure 3:**
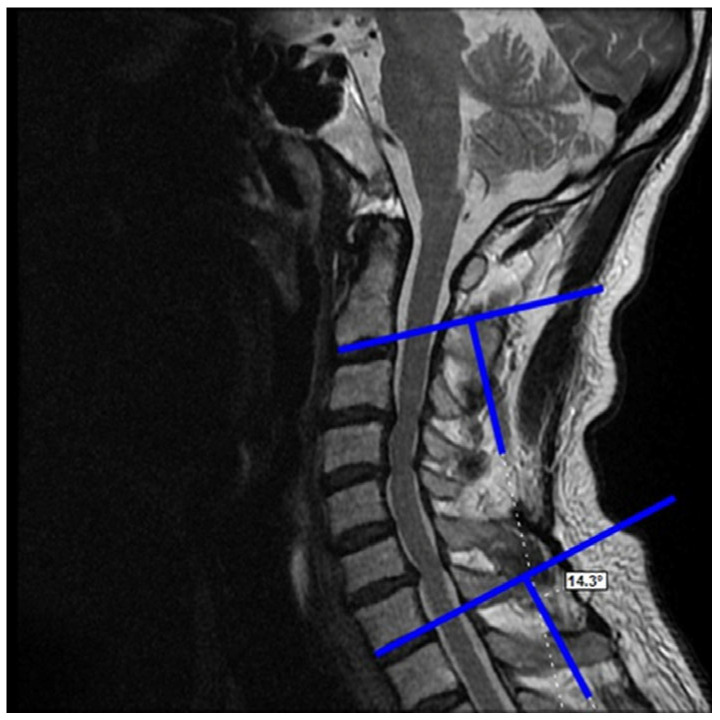
Evaluation of cervical lordosis angle, as the angle measured between the line drawn along the inferior endplate of C2 and the line drawn along the inferior endplate of C7.

**Figure 4:**
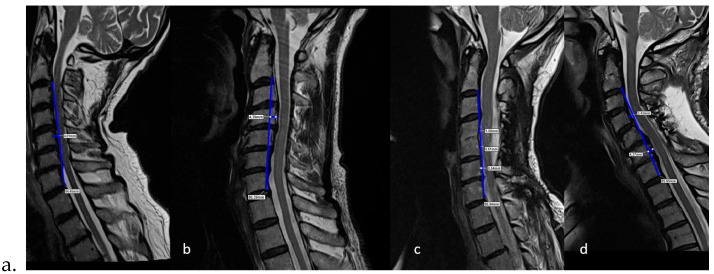
Evaluation of cervical alignment type: (**a**) Lordosis—none of the posterior margins of the C3, C4, C5, and C6 vertebrae are located behind the blue line, and at least one vertebra (C5) lies at least 2 mm in front of it. (**b**) Kyphosis—none of the posterior margins of the C3, C4, C5, and C6 vertebrae are located in front of this blue line, and at least one vertebra (C4) lies at least 2 mm behind it. (**c**) Straight—the posterior margins of the C3, C4, C5, and C6 vertebrae are aligned on, in front of, or behind the blue line, but none are at least 2 mm in front of or 2 mm behind it. All are positioned close to the line. (**d**) Sigmoid—at least one of the C3, C4, C5, or C6 vertebrae lies at least 2 mm in front of blue line (C6), and at least one (C4) lies at least 2 mm behind it.

**Figure 5:**
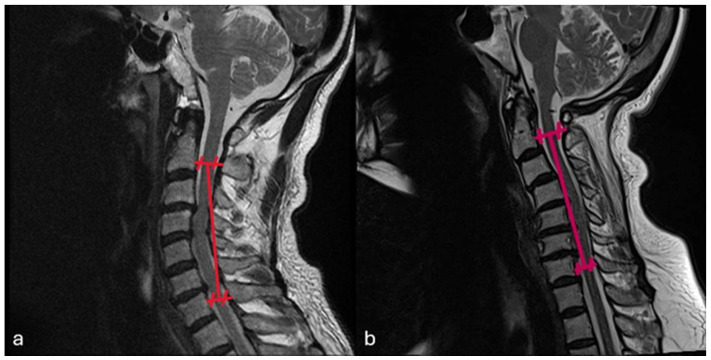
Evaluation of K-line: (**a**) K-line +; (**b**) K-line −.

1. C2–C7 Cobb angle (Figure 3)
2. Cervical alignment (Figure 4)
3. K-line (Figure 5)

#### 2.2.3. Intraoperatively Identified Independent Variables

1. Lamina lifting side
2. Total number of levels applied laminoplasty
3. Whether laminoplasty performed on C3 or not

#### 2.2.4. Independent Variables Detected on Early Postoperative CT Imaging

**Figure 6:**
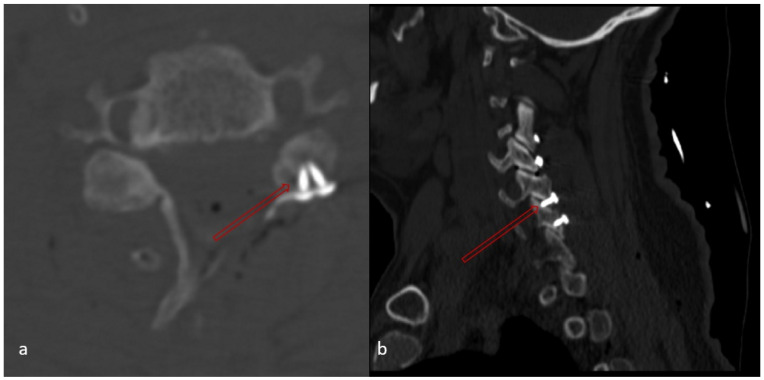
Facet joint disturbance at the C5–6 level: (**a**) red arrow shows screws on lateral mass disturbing facet joint on axial CT; (**b**) red arrow shows facet joint disturbance on sagittal CT.

1. Whether there is a lamina fracture or not
2. Total number of lamina fractures
3. Whether or not there is a fracture at the highest level where laminoplasty was performed
4. Whether any of the screw placed in the lateral mass causes facet joint disturbance or not (Figure 6),
5. Number of facet disturbance,
6. Whether there is uppermost facet disturbance or not,
7. Whether there is lowermost facet disturbance or not

Cervical alignment was evaluated by drawing a line from the posterior–inferior corner of the C2 vertebral body to the posterior–inferior corner of the C7 vertebral body and measuring the distance to the midpoint of the posterior border of the vertebral body. Alignment was divided into four different groups: lordotic, kyphotic, straight, and sigmoid [10] (Figure 4).

The K-line was drawn as a straight line connecting the midpoints of the spinal canal at C2 and C7. If the anterior structures do not intersect the K-line, the case is defined as K-line positive (+); if some of the anterior structures pass posterior to the K-line, it is defined as K-line negative (−) (Figure 5).

### 2.3. Dependent Variables (Radiological)

1. Postoperative last control C2–C7 Cobb angle
2. Postoperative last control cervical alignment
3. Postoperative last control K-line

Preoperative radiological assessment was defined as the most recent imaging obtained within one month prior to surgery. Postoperative radiological assessment referred to the most recent imaging acquired at least one month after surgery.

### 2.4. Observer Agreement

First, 30 cases were randomly selected from the entire cohort for the assessment of interobserver reliability. Three neurosurgeons (I.E.S., S.B., E.E.) independently evaluated the cervical lordosis angle in each case from lateral cervical radiographs, CT, and MRI images. Intraclass correlation coefficients (ICCs) were calculated for the Cobb angle measurements across the three imaging modalities. After confirming adequate interobserver agreement, the remaining radiological examinations were divided among the three neurosurgeons for further measurements. To assess intraobserver reliability, the same 30 cases were re-evaluated by each neurosurgeon four weeks after the initial assessment under blinded conditions. ICC values were calculated for each modality to determine intraobserver agreement. The ICC values were interpreted as follows: ≥0.90, excellent; 0.75–0.89, good; 0.50–0.74, moderate; and <0.50, poor. Furthermore, Bland–Altman analysis was conducted to complement the agreement assessments between modalities. Mean differences and 95% limits of agreement were calculated and are illustrated using Bland–Altman plots.

### 2.5. Statistical Analysis

The data were analyzed using the SPSS, Version 30.0 (IBM Corp., Armonk, NY, USA) Inter- and intraobserver measurement reliability for both preoperative and postoperative MRI assessments was evaluated using the intraclass correlation coefficient (ICC). Agreement between raters was additionally assessed using Bland–Altman plots to visually evaluate measurement bias and limits of agreement. Descriptive statistics were calculated for all preoperative and postoperative variables. Comparisons were conducted using the chi-square test for nominal variables, the independent sample *t*-test for normally distributed scale variables, and the Mann–Whitney U-test for non-normally distributed scale variables.

Variables demonstrating significance in univariate analysis (*p* < 0.10) were subsequently entered into a multivariate logistic regression model to determine independent predictors of postoperative loss of cervical lordosis. Odds ratios (ORs) with corresponding 95% confidence intervals (CI) were calculated. A *p*-value < 0.05 was considered statistically significant.

## 3. Results

### 3.1. Observer Reliability

**Figure 7:**
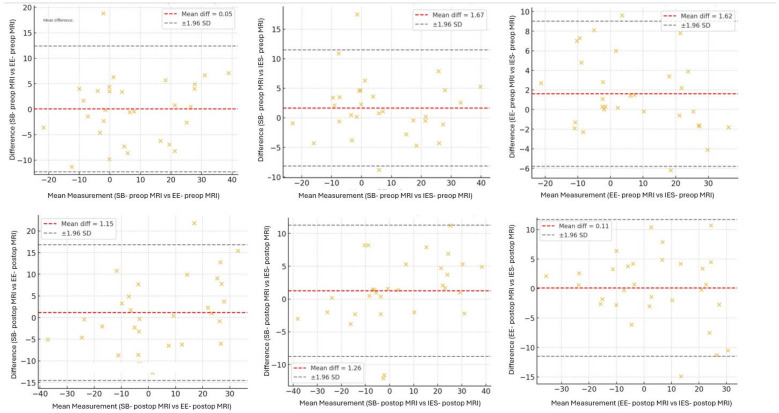
Bland–Altman plots assessing inter-observer agreement for preoperative and postoperative MRI. Each plot shows the difference between measurements performed by two independent observers plotted against their mean value. Red dashed lines indicate mean bias; grey dashed lines represent ±1.96 SD.

Interobserver agreement was excellent for both preoperative and postoperative MRI measurements, and intraobserver reliability remained stable and excellent after a four-week re-assessment interval. Corresponding ICC values are presented in Table 1, and Bland–Altman plots of interobserver agreement are shown in Figure 7.

### 3.2. Study Population

A total of 256 patients who underwent surgery with the open-door laminoplasty technique were reviewed for this study. Fifteen patients with a previous history of cervical anterior surgery, six patients operated on due to trauma, one patient who underwent an operation for cervical abscess, one patient with Klippeil–Feil syndrome, one patient with lateral mass screw insertion, and one patient with a C3 laminectomy were excluded from the study. The pre- and postoperative (at least one month after surgery) distribution of available imaging studies was as follows: Direct radiography (13/16), CT (217/86), and MRI (174/100). The numbers of patients with both pre- and postoperative imaging of the same type for comparison were as follows: 3 with plain radiography, 76 with CT, and 78 with MRI.

To ensure accurate comparisons of cervical lordosis and other sagittal parameters, measurements were performed using the same imaging modality in both periods. Among all patients, 78 individuals had both preoperative and postoperative MRI scans available and, therefore, constituted the study cohort for the final analysis. The average time from surgery to the last postoperative MRI control was 481 ± 366 (min: 52, max: 1274) days.

All three dependent radiological variables—C2–C7 Cobb angle, cervical alignment category, and K-line status—were obtained from postoperative MRI. Their corresponding preoperative values were also derived from preoperative MRI of the same patients. Thus, the analysis was performed exclusively on the 78 patients with complete and comparable MRI data.

The average age of these 78 cases was 56.5 ± 11.2 years. The gender distribution consisted of 45 males (57.7%) and 33 females (42.3%).

### 3.3. Relationships Between Independent and Dependent Variables

#### 3.3.1. Preoperative Demographic Independent Variables vs. Dependent Variables

Both age (*p* = 0.344) and gender (*p* = 0.403) did not significantly affect the development of postoperative kyphosis.

#### 3.3.2. Preoperative Radiological Independent Variables vs. Dependent Variables

##### Preoperative C2–C7 Cobb Angle vs. Postoperative Last Control C2–C7 Cobb Angle

The mean preoperative cervical Cobb angle was 8.78 ± 13.75 degrees, while the mean postoperative angle was 6.49 ± 13.82 degrees, indicating a statistically significant reduction (paired *t*-test, *p* = 0.024).

When patients were divided into two groups based on their preoperative C2–C7 Cobb angles—namely, as low (less than or equal to 10 degrees) and high (more than 10 degrees)—the lordosis of those with low C2–C7 Cobb angles changed by an average of −0.09 ± 8.87 degrees in the postoperative period, while those with high C2–C7 Cobb angles changed by an average of −4.99 ± 8.06 degrees; this difference was significant (Independent sample *t*-test, *p* = 0.013).

##### Preoperative Cervical Alignment Category vs. Postoperative Cervical Alignment Category

Patients mostly maintained their preoperative alignment in the postoperative period. Among the 36 patients with preoperative lordotic alignment, 29 preserved lordosis, whereas 1 developed kyphosis and 6 shifted to a straight pattern. Conversely, of the 18 patients with preoperative kyphotic alignment, 13 remained kyphotic, 4 shifted to straight, and 1 transitioned to lordosis (Table 2).

##### Preoperative K-Line vs. Postoperative K-Line

Among the four patients who were operated on with a preoperative negative K-line, the K-line status persisted postoperatively as negative in three patients, whereas it transitioned to positive in one patient. While the K-line of 72 of the 74 patients who underwent surgery as K-line (+) remained as (+) at the last postoperative follow-up, two patients exhibited a change to K-line (−).

#### 3.3.3. Intraoperatively Identified Independent Variables vs. Dependent Variables

##### Whether or Not Laminoplasty Performed on C3 vs. Postoperative Last Control C2–C7 Cobb Angle

In cases where the C3 vertebra was included in laminoplasty (61 cases), the cervical lordosis angle changed by −3.42 ± 8.32 degrees. In comparison, in cases without C3 involvement (17 cases), the cervical lordosis angle changed by +1.77 ± 9.58 degrees. Inclusion of the C3 level in laminoplasty was found to be a significant factor affecting cervical lordosis (independent samples *t*-test, *p* = 0.031).

#### 3.3.4. Independent Variables Detected on Early Postoperative CT Imaging vs. Dependent Variables

All 78 patients underwent early postoperative computed tomography (CT) scans.

##### Whether There Is a Lamina Fracture vs. Postoperative Last Control C2–C7 Cobb Angle 

There was a statistically significant relationship between lamina fracture and the change in the cervical lordosis angle. The cervical lordosis angle decreased more when there was a lamina fracture during surgery than when there was no lamina fracture (independent sample *t*-test, *p* = 0.013). In cases with lamina fractures (40 cases), the cervical lordosis angle changed by −4.67 ± 9.53 degrees; in contrast, in cases without lamina fractures (38 cases), this change was +0.21 ± 7.30 degrees.

A fracture at the lowest level of laminoplasty did not significantly affect the development of postoperative kyphosis. (*p* = 0.226).

##### Whether or Not There Is a Uppermost Lamina Fracture vs. Postoperative Last Control C2–C7 Cobb Angle 

The presence of a lamina fracture at the highest laminoplasty level was significantly associated with greater loss of cervical lordosis (Independent samples *t*-test, *p* = 0.004). Patients with a cranial-most lamina fracture showed a mean change of −7.09 ± 9.74, compared with −0.63 ± 7.90 in patients without such fractures.

##### Whether or Not There Is Uppermost Facet Disturbance vs. Postoperative Last Control C2–C7 Cobb Angle 

A significant relationship was found between the presence of facet joint disturbance at the uppermost vertebral level where laminoplasty was performed and the change in the cervical lordosis angle. When facet disturbance was present at the uppermost level where laminoplasty was performed (29 cases), cervical lordosis decreased by −5.11 ± 9.30 degrees. By contrast, in instances without uppermost facet disturbance (49 cases), the cervical lordosis change was −0.62 ± 8.15 degrees (Independent samples *t*-test, *p* = 0.036).

Disturbance in any facet joint (*p* = 0.390) or in the lowest-level facet joint (*p* = 0.147) did not significantly affect the development of postoperative kyphosis.

### 3.4. Logistic Regression Analysis

To further identify independent risk factors for postoperative loss of cervical lordosis, a logistic regression analysis involving demographic, radiological, and surgical variables was performed. Among all tested predictors, only the uppermost facet disturbance was found to significantly increase the risk of lordosis loss. Other variables, including age, sex, total number of operated levels, C3 involvement, and laminar fractures, did not demonstrate independent predictive value. The detailed results of the logistic regression analysis are presented in Table 3 and Table 4.

## 4. Discussion

This study demonstrates that uppermost facet joint disturbance is the key independent determinant of postoperative cervical alignment after open-door laminoplasty, increasing the risk of cervical lordosis loss. Despite our initial results, cranial lamina fracture did not retain significance in the multivariate model, suggesting that its previously observed effect may be secondary to concomitant facet disruption.

### 4.1. Uppermost Facet Disturbance: The Key Independent Predictor

Among all operative variables, disturbance of the cranial-most facet joint emerged as the single strongest risk factor for postoperative loss of lordosis. Although earlier studies have linked general facet violations to inferior outcomes [11,12], our findings refine this relationship by demonstrating that not all facet injuries carry equal risk. The uppermost facet—typically the C2–3 or C3–4 joint, depending on surgical extent—acts as the proximal anchor of the posterior tension band. Even minor mechanical disruptions at this level compromise the extension moment and reduce the stabilizing resistance provided by the semispinalis cervicis and multifidus muscle insertions.

Min et al. defined optimal screw trajectories to avoid facet violation [13], while Chen et al. described safe zones for lateral mass screw placement [14]. Our results support these recommendations by emphasizing that protection of the uppermost facet is especially critical, given that its violation has a disproportionate biomechanical impact. Therefore, careful trajectory planning and the use of a different screw size or obliquely directed screws should be prioritized at this level. A caudal entry point for lateral mass screw placement is, in our opinion, the primary factor contributing to facet joint violation. To minimize this risk, the screw entry point should be positioned closer to the inferior articular process of the cranial vertebra, rather than the caudal aspect of the lateral mass.

Additionally, inserting the screws with a cranially oriented trajectory—rather than a perpendicular or caudally directed angle relative to the cervical axis—may further help to prevent iatrogenic facet joint encroachment.

Importantly, the absence of a significant correlation between overall facet disturbance count and cervical lordosis angle change indicates that only the uppermost facet exerts meaningful biomechanical influence. This suggests that the cranial facet functions as a mechanical keystone for maintaining cervical lordosis, while caudal facets contribute less to sagittal balance.

### 4.2. Role of Preoperative Lordosis

Although preoperative cervical lordosis did not emerge as a statistically independent predictor of postoperative sagittal change in the regression analysis, the alignment transition patterns observed in our cohort provide clinically meaningful insights; in particular, most patients tended to maintain their original sagittal alignment after surgery.

Importantly, postoperative sagittal alignment changes were not unidirectional. In addition to patients transitioning from lordotic to straight or kyphotic alignment, some patients demonstrated alignment improvement from kyphotic or straight to straight or lordotic profiles. This bidirectional pattern suggests that postoperative alignment changes are unlikely to be solely attributable to an inferior lordosis-preserving capacity of laminoplasty and are more likely influenced by individual patient-related factors.

Patients with initially higher lordosis still exhibited larger absolute reductions postoperatively, a finding that can be explained by a “ceiling effect”: those who begin with greater lordosis have more range to lose, yet they remain less likely to transition to kyphosis as their baseline geometry provides a safety margin. This aligns with prior showing that smaller baseline angles predispose patients to postoperative deformity [6,15,16].

### 4.3. Uppermost Lamina Fracture: Loss of Significance in Multivariate Analysis

Although the univariate analysis associated cranial lamina fracture with postoperative loss of lordosis, this variable lost statistical significance after adjustment. This attenuation implies that lamina fracture per se may not directly destabilize the cervical spine but rather acts as a surrogate marker for increased mechanical stress or technical inaccuracy that concurrently injures the facet joint. Previous reports have described hinge fractures as contributors to kyphotic drift [11,17]; however, our findings suggest that facet violation—and not laminar fracture alone—is the critical link in this cascade. Consequently, intraoperative attention should be focused on preserving the hinge side of the laminoplasty continuity without transmitting force to the facet capsule, especially at cranial levels.

### 4.4. Role of C3 Involvement

Although C3 involvement was associated with greater postoperative loss of lordosis in the univariate analysis, it did not remain significant in the multivariate logistic regression. This attenuation likely reflects collinearity between C3 involvement and uppermost facet joint disturbance: laminoplasty extended to C3 frequently necessitates wider cranial exposure, increasing the likelihood of C2–3 facet complex disruption. Thus, the observed risk attributed to “C3 involvement” in univariate testing appears to derive not from the anatomical level itself, but from the accompanying cranial facet compromise that commonly occurs when the laminoplasty reaches C3.

Therefore, when the C2–3 facet joint is preserved, including C3 in the laminoplasty range does not independently increase the risk of postoperative kyphosis. This explanation also clarifies why previous studies have reported conflicting results: some observed an association between C3 involvement and loss of lordosis, whereas others found that this association disappeared after controlling for facet integrity and preservation of adjacent structures [11,17].

This interpretation is further supported by previous studies demonstrating that cranial extension of laminoplasty increases susceptibility to proximal facet compromise and subsequent loss of alignment—particularly at the C2–3 junction—as consistently highlighted in biomechanical, radiological, and large-cohort alignment analyses [13,15,18].

### 4.5. Clinical Implications and Limitations

The presented findings reinforce the concept that proximal surgical integrity is a more decisive determinant of postoperative alignment than global preoperative morphology. Maintaining the uppermost facet joint, protecting the C2–3 posterior tension band, and minimizing disruption of muscle insertions (notably, the semispinalis cervicis at C2) are practical strategies for preserving lordosis. This recognition provides surgeons with practical strategies to minimize the loss of lordosis.

The limitations of the study must also be acknowledged. The reliance on supine MRI can lead to underestimation of physiological lordosis when compared with standing radiographs, potentially influencing the external validity. However, the uniform use of MRI across all patients ensured consistency for internal comparisons. In addition, the preference for MRI-based follow-up may be advantageous due to the avoidance of repeated exposure to ionizing radiation associated with serial standing radiographs, in which visualization of the C7 vertebral body is frequently limited by shoulder superimposition.

Our previous study established a validated conversion model between supine MRI and standing radiographs [19], defined by the following equation: Estimated X-ray Cobb angle = 7.59 + 0.71 × (MRI Cobb angle). This formula confirms that MRI reliably predicts upright lordosis and safely excludes true kyphosis when the MRI Cobb angle exceeds 1.2°, supporting the methodological validity of MRI-based measurements in the present study.

Comorbidities such as osteoporosis could not be analyzed, as bone mineral density assessments and formal osteoporosis diagnoses were not routinely available in the retrospective clinical records of patients undergoing cervical laminoplasty.

Despite these limitations, the novel identification of uppermost facet disturbance as an independent risk factor provides a unique and clinically actionable contribution to the literature.

## 5. Conclusions

The results of this study highlight that postoperative kyphosis after cervical laminoplasty is determined not only by baseline cervical lordosis but also—and more importantly—by surgical integrity at the proximal cranial level. We identified a novel and clinically actionable risk factor—uppermost facet disturbance—which exerted a disproportionate influence on loss of lordosis. Alongside established variables such as preoperative lordosis and C3 involvement, these findings underscore the critical role of meticulous cranial-level preservation in maintaining postoperative alignment. Recognition of these factors offers surgeons practical strategies for the reduce kyphotic change and optimization of long-term outcomes. Future prospective multicenter studies are warranted to validate these results and refine risk stratification models integrating both global and cranial-level predictors.

## Figures and Tables

**Table 1:** Interobserver and intraobserver reliability results.

Imaging	Interobserver Reliability *	Intraobserver Reliability *		
		SB	IES	EE
**Preop-MRI**	0.981	0.940	0.910	0.946
**Postop-MRI**	0.940	0.951	0.903	0.910

* ICC values.

**Table 2:** Preoperative and postoperative alignment categories.

	Preoperative MRI Alignment				
	Lordosis	Straight	Sigmoidal	Kyphosis	Total
**Postoperative MRI Alignment**					
**Lordosis**	29	2	0	1	32
**Straight**	6	13	0	4	23
**Sigmoidal**	0	1	1	0	2
**Kyphosis**	1	7	0	13	21
**Total**	36	23	1	18	78

**Table 3:** Univariate analysis of risk factors for postoperative loss of cervical lordosis.

Factors	OR	95% CI	*p*
**Age**	1.00	0.97–1.05	0.723
**Gender (female)**	0.52	0.21–1.29	0.160
**Preop Cobb angle**	1.01	0.98–1.5	0.321
**Preop alignment (lordosis)**	0.93	0.62–1.38	0.726
**Level number of laminoplasty**	1.72	0.92–3.23	**0.091**
**C3 laminoplasty**	2.36	0.79–7.06	0.124
**Lamina fracture**	1.86	0.74–4.60	0.182
**Lamina fracture at the top level**	2.80	0.89–8.71	**0.075**
**Lamina fracture at the lowest level**	1.63	0.54–4.93	0.382
**Facet disturbance**	3.00	0.89–10.00	**0.073**
**Facet disturbance at the top level**	2.73	1.01–7.34	0.046
**Facet disturbance at the lowest level**	2.53	1.00–6.37	**0.048**

**Table 4:** Multivariate analysis of risk factors for postoperative loss of cervical lordosis.

Factors	OR	95% CI	*p*
**Level number of laminoplasty**	1.24	0.78–2.11	0.297
**Lamina fracture at the top level**	1.63	0.56–4.83	0360
**Facet disturbance**	1.18	0.40–3.42	0.761
**Facet disturbance at the top level**	**4.62**	1.07–19.3	**0.039**
**Facet disturbance at the lowest level**	1.72	0.65–4.51	0.273

## Data Availability

The data presented in this study are available from the corresponding author upon reasonable request, in accordance with ethical and privacy restrictions.

## Abbreviations

MRI	Magnetic resonance imaging
CT	Computed tomography
CSM	Cervical spondylotic myelopathy
OPLL	Ossification of the posterior longitudinal ligament
ICC	Intraclass correlation coefficient
